# MOXD1 knockdown suppresses the proliferation and tumor growth of glioblastoma cells via ER stress-inducing apoptosis

**DOI:** 10.1038/s41420-022-00976-9

**Published:** 2022-04-07

**Authors:** Pengfei Shi, Jie Xu, Fanwei Xia, Yinggang Wang, Jie Ren, Ping Liang, Hongjuan Cui

**Affiliations:** 1 State Key Laboratory of Silkworm Genome Biology, Southwest University, 400716 Chongqing, China; 2 Cancer Center, Medical Research Institute, Southwest University, 400716 Chongqing, China; 3 Department of Neurosurgery, Children’s Hospital of Chongqing Medical University, National Clinical Research Center for Child and Disorders, Ministry of Education Key Laboratory of Child Development and Disorders, Chongqing, 400014 China; 4 Chongqing Key Laboratory of Pediatrics, Chongqing, 400014 China

**Keywords:** CNS cancer, Cancer epigenetics

## Abstract

Oxygenase-catalyzed reduction and activation of oxygen molecules and the incorporation of oxygen atoms into organic molecules are undoubtedly necessary in the process of tumor development, and it is also one of the research hotspots in recent years. MOXD1 belongs to the copper-dependent monooxygenase family. The expression of MOXD1 is one of the characteristics of early tumor development. However, it is not understandable that the biological function and molecular mechanism of MOXD1 in Glioblastoma (GBM). In this study, high MOXD1 expression is strongly associated with poor survival of the patient with GBM. Moreover. MOXD1 knockdown can inhibit cell viability, proliferation, migration, invasion, and tumorigenesis of GBM cells. This is also proven for the first time that MOXD1 can bind to β3GnT2 and affect the glycosylation modification of some proteins. In addition, knockdown of MOXD1 induces endoplasmic reticulum (ER) stress and triggers the ER–mitochondrial apoptosis pathway. Taken together, these results reveal that MOXD1 is involved in the occurrence and development of GBM, and also provide a new strategy for targeted therapy.

## Introduction

Glioblastoma (GBM) is the most common primary malignant tumor of the central nervous system. The characteristics of this malignant tumor include uncontrolled cell proliferation, invasiveness with long root-like protrusions and single invasive cells, areas of necrosis, resistance to apoptosis, extensive angiogenesis, and the ability to alter multiple genetic genes [1]. At present, the clinical treatment of GBM is mainly surgical resection, radiation therapy and temozolomide chemotherapy, but due to the neurotoxicity of the drug (dose limitation), it is difficult to reach the central nervous system through the blood–brain barrier [2]. The medicinal properties and the complete surgical resection cannot be achieved due to the aggressiveness of the disease. Therefore, a lot of basic and clinical research has been carried out in the past few decades, but little progress has been made in the prevention and treatment of this devastating disease, indicating that a more in-depth study of the growth mechanism of GBM is needed.

Monooxygenase DBH like 1 (MOXD1) is a member of the copper monooxygenase family, including dopamine monooxygenase (dopamine β-monooxygenase, DBM) and peptidylglycine α-hydroxylating monooxygenase (PHM), located on chromosome 6, is a protein-coding gene with the highest expression in salivary glands and ovaries, and moderate levels in brain, pituitary and heart [3]. MOXD1 is located on the endoplasmic reticulum (ER) of endocrine or non-endocrine cells [4]. MOXD1 firstly is discovered when looking for genes whose expression changes in aging human fibroblasts. MOXD1 transcripts are also detected at the boundary of the neural plate (stage 7), expressed during the development of the neural crest, and distributed along the olfactory bulb, cerebellum, brain stem, and parietal cortex with the development of nerve cells, participating in the basal plate. The formation of neuronal cells is a marker of the dual states of the anterior medial region, the final striate nucleus, and the medial amygdala [5]. The clinical database shows that MOXD1 is highly expressed in GBM cells and can promote the growth of cancer cells. Moreover, the expression of MOXD1 is one of the characteristics of early gastric cancer [6].

Studies have shown that N-acetylglucosaminyltransferase-I (GnT-I) are involved in numerous biologic processes, such as cell adhesion, migration, and invasion [7]. Expression of N-Acetylglucosaminyltransferase III (-IGnTII) promotes trophoblast invasion and migration in early human placenta, and GnT-III also participates in tumor invasion and metastasis [8]. N-Acetylglucosaminyltransferase-II (GnT-II) increases matrix metalloproteinase 2 activity and promotes tumorigenicity in neuroblastoma cell line [9]. β-1,3-N-acetylglucosaminyltransferase-T2 (β3GnT2) is a differentially expressed novel gene sharing structural similarity with the human β3-galactosyltransferase family. Moreover, β3GnT2 is the major poly-N-acetyl-lactosamine synthase, and deletion of its coding gene dramatically reduced the cell surface poly-N-acetyl-lactosamine and led to hypersensitive and hyperresponsive immunocytes. Therefore, β3GnT2 is an important player in immune homeostasis [10]. Overexpression of β3GnT2 is involved in breast cancer cell growth [11]. This indicates β3GnT2 involve in human cancer progression [12].

In this study, we demonstrated that MOXD1 can interact with β3GnT2, and affect proliferation, migration, and invasion of GBM cells. In addition, knockdown of MOXD1 using lentiviral transfection biotechnology can activate the ER stress–mitochondrial apoptosis pathway to suppress the growth of GBM cells in vitro and in vivo.

## Results

### High MOXD1 expression is with poor prognosis in patients with GBM

**Fig. 1:**
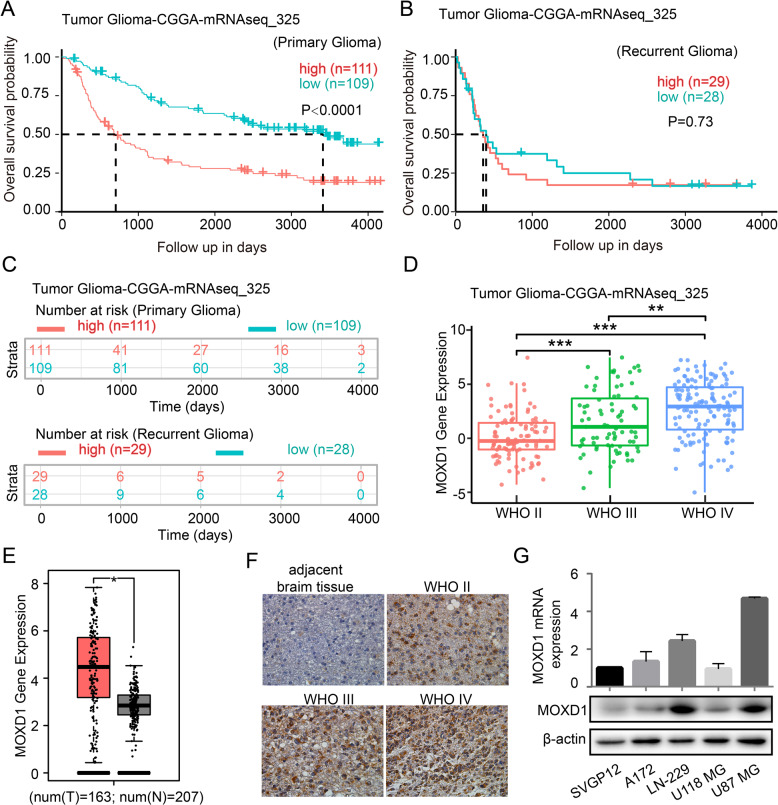
High MOXD1 expression is with poor prognosis in patients with glioblastoma. **A**–**C** Results of the Kaplan–Meier analysis of progression-free survival and the log-rank test *p* values are indicated for tumor glioma-CGGA-mRNAseq-325. **D** Box plot of MOXD1 expression levels in grade II–IV gliomas in tumor glioma-CGGA-mRNAseq-325. **E** Box plot of MOXD1 expression levels in normal tissues and GBM in tumor glioma-GEPIA-analysis. **F** Immunohistochemistry staining of patient clinical GBM samples. **G** The relative levels of MOXD1 in five cell lines (SVGP12, A172, LN-229, U118MG, and U87MG cells) were detected by qRT-PCR and Western blot.

To determine whether MOXD1 is related to poor survival in patients with primary GBM, we analyzed the survival data form the databases (Tumor Glioma-CGGA-mRNAseq-325 and Tumor Glioma-French-284). The databases showed that high MOXD1 expression is strongly associated with poor survival (Fig. 1A and Supplementary Fig. 1A). However, the expression of MOXD1 is not significantly different from the recurrence of GBM patients (Fig. 1B). Moreover, the expression of MOXD1 is not significantly different from number at risk in primary GBM or recurrent GBM (Fig. 1C). Kaplan–Meier analysis indicated that MOXD1 expression level was positively associated with GBM grade (Fig. 1D). Columnar analysis also showed that MOXD1 in GBM tissue was higher than that in adjacent normal tissues (Fig. 1E). Moreover, the expression of MOXD1 in IDH wildtype status and 1p/19q non-codel status is significantly higher the IDH mutation status and 1p/19q codel status (Supplementary Fig. 1B, C). The expression of MOXD1 is also related to the patient’s age, but not to gender (Supplementary Fig. 1D, E). The section of the patient’s tissue also showed MOXD1 expression level was positively associated with GBM grade (Fig. 1F). Next, qRT-PCR and Western blotting were used to detect the expression of MOXD1 in in several GBM cell lines and astrocytes. The expression of MOXD1 in LN-229 and U87 MG is higher than other cell lines (Fig. 1G).

### Blocking MOXD1 inhibits GBM cell proliferation and induces cell arrest

**Fig. 2:**
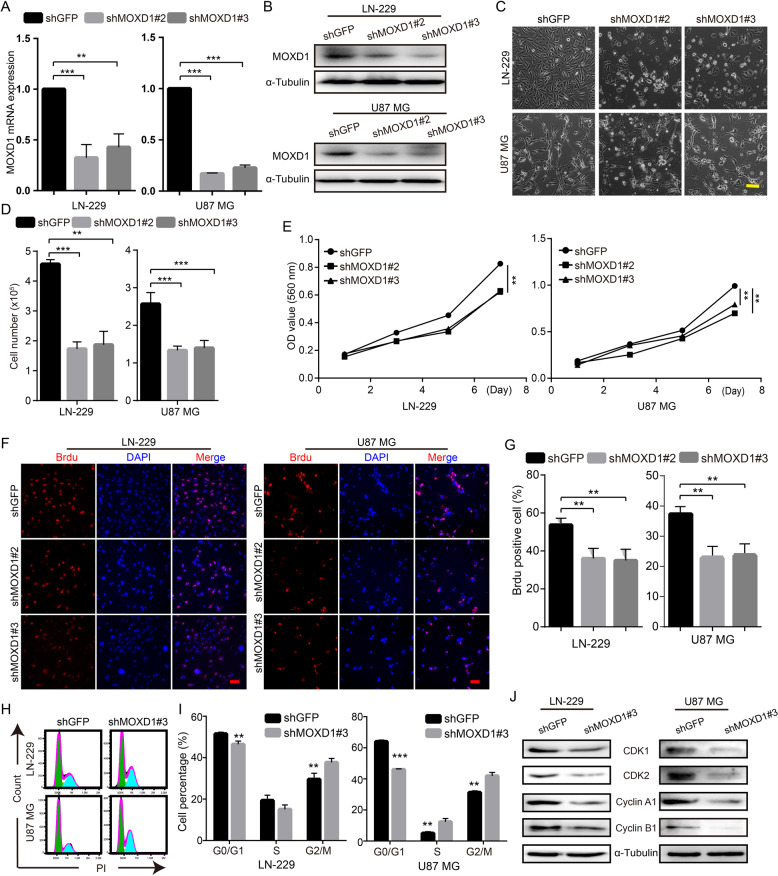
Blocking MOXD1 inhibits GBM cell proliferation and induces cell arrest. **A** After MOXD1 knockdown by shRNA in GBM cell lines (LN-229, U87 MG), MOXD1 expression was detected using qRT-PCR. **B** After MOXD1 knockdown by shRNA in GBM cell lines (LN-229, U87 MG), MOXD1 expression was detected using Western blot analysis. **C** One thousand cells were tiled in a six-well plate and the morphology of the cells was observed after culturing for 48 h. **D** One thousand cells are flattened into a six-well plate, and the number of cells is counted after culturing for 48 h. **E** MOXD1 knockdown inhibited the proliferation of LN-229 and U87 MG cells, MTT assay was used to examine the effect of MOXD1 knockdown on cell viability. **F** BrdU assays was used to detect the DNA synthesis ability after MOXD1 knockdown. **G** The statistics of the amount of DNA synthesis of LN-229 and U87 MG cells ability after MOXD1 knockdown. **H** Cell cycle was assessed by flow cytometry after MOXD1 knockdown. **I** Statistics of each cycle of the cell after MOXD1 knockdown. **J** Western blot assay was executed to detect the expression of G2/M cell cycle regulatory proteins in MOXD1 knockdown cells.

To explore the effect of MOXD1 on GBM cell proliferation, we knocked down MOXD1 by two independent short hairpin RNA (shRNA) sequences against MOXD1 in GBM cell lines (LN-229 and U87 MG), and this two shRNA were named shMOXD1#2 and shMOXD1#3. QRT-PCR analysis and Western blot analysis showed that shMOXD1#2 and shMOXD1#3 can significantly reduce the RNA level and protein level of MOXD1 (Fig. 2A, B). We culture one hundred thousand cells in a six-well plate. After 48 h of cultivation, we observed that shMOXD1#2 and shMOXD1#3 can inhibit GBM cell proliferation (Fig. 2C, D). 3-[4,5-dimethylthiazol-2-yl]-2,5 diphenyl tetrazolium bromide (MTT) assays also declared that shMOXD1 can reduce cell proliferation rate (Fig. 2E). Moreover, BrdU assays further showed that MOXD1 knockdown resulted in a significant reduction in DNA synthesis (Fig. 2F, G). Then, we tested the cell cycle of control cells and MOXD1 knockdown cells by flow cytometry, and found that MOXD1 knockdown can induce cell cycle arrest at the G2/M phase (Fig. 2H, I). In order to understand the molecular mechanisms underlying of MOXD1 knockdown-induced cell cycle arrest, we discovered several G2/M phase related proteins. We found that MOXD1 knockdown can reduce the expression of CDK1, CDK2, Cyclin A1 and Cyclin B1 (Fig. 2J). In short, MOXD1 is involved in the growth and proliferation of GBM.

### Blocking MOXD1 inhibits self-renewal and tumor growth of GBM

**Fig. 3:**
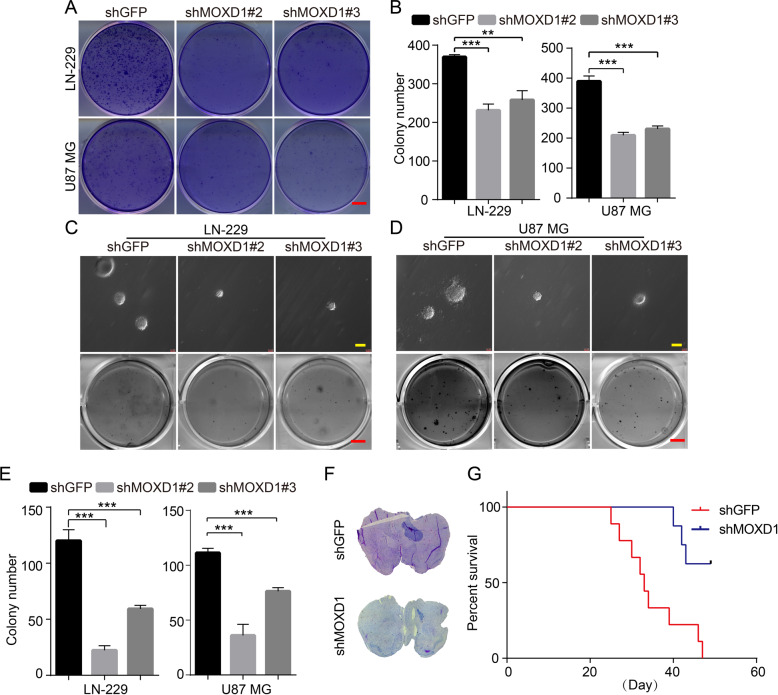
Blocking MOXD1 inhibits self-renewal and tumor growth of GBM. **A** One thousand cells were tiled in a six-well plate and the planar cloning assay was used to detect the self-renewal ability in MOXD1 knockdown cells. **B** Statistics of cell self-renewal after culturing for 7 days. **C**, **D** Soft agar assays was used to detect the self-renewal ability in MOXD1 knockdown cells. **E** Statistics of cell spheroidizing ability after culturing for 20 days. **F** HE staining was used to detect tumorigenesis of GBM intracranial injection in mice. **G** Survival curve of mice injected intracranially with MOXD1 knockdown cells.

In order to explore the effect of MOXD1 on tumor cell self-renewal and tumor formation, we first performed a planar cloning experiment, and the result showed that MOXD1 knockdown could significantly inhibit the planar cloning ability of GBM cells (Fig. 3A, B). Next, soft agar assays further indicated that the knockdown of MOXD1 inhibited the colonies of GBM cells (Fig. 3C–E). We then established a xenograft model to further assess the effects of MOXD1 on GBM cell self-renewal in vivo. The result showed that the size of GBM in the brain of mice injected with shMOXD1 GBM cells was significantly smaller than that of mice injected with shGFP GBM cells, and the survival time of mice injected with shMOXD1 GBM cells was significantly longer than that of mice injected with shGFP GBM cells (Fig. 3F, G).

### Blocking MOXD1 prevents migration and invasion of GBM cells

**Fig. 4:**
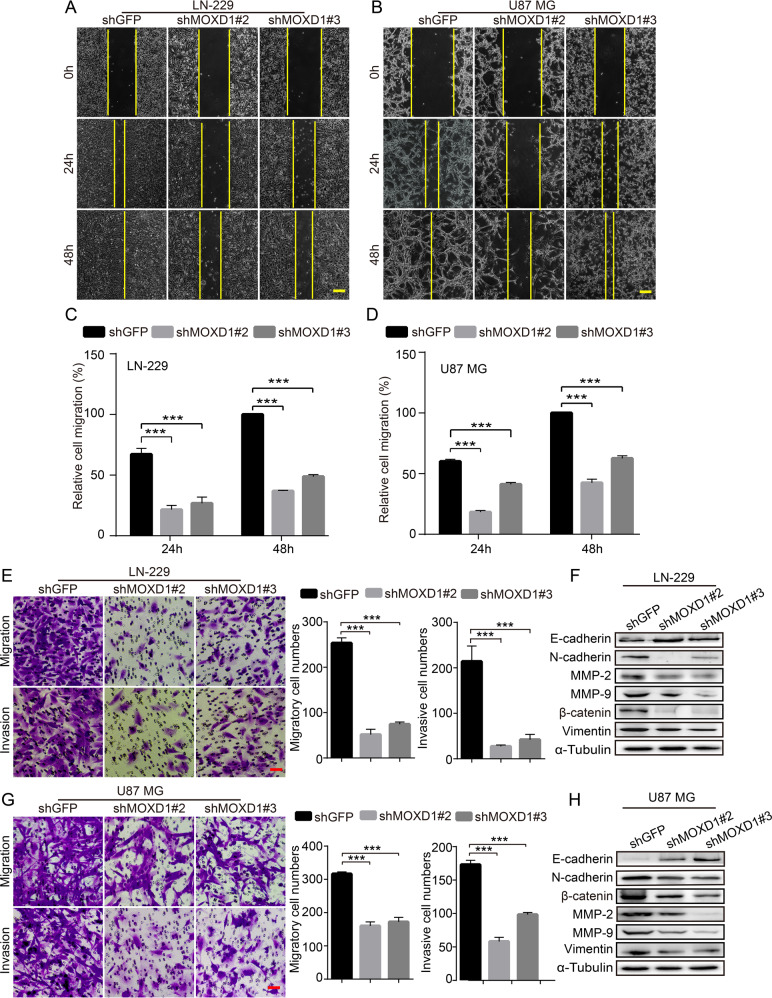
Blocking MOXD1 prevents migration and invasion of GBM cells. **A**, **B** Scratch test was used to detect cell migration distance after MOXD1 knockdown cells. **C**, **D** Statistics of migration distance after MOXD1 knockdown cells. **E**, **F** Migration and invasion assays were performed to detect the effect of MOXD1 knockdown on LN-229 and U87 MG cells. **F**, **H** EMT-related proteins are detected in MOXD1 knockdown cells.

As the most aggressive brain tumor, GBM cells have a strong ability to migration and invasion. Therefore, to explore whether MOXD1 affects cell migration and invasion of GBM, we conducted firstly a scratch experiment. The results showed that the knockdown of MOXD1 significantly delayed the confluence rate of GBM cells (Fig. 4A–D). Then we used transwell and matrigel to further explore the effect of MOXD1 on GBM cell migration and invasion. We found that the knockdown of MOXD1 significantly reduced the cell numbers of invasion and migration (Fig. 4E, G). We further found that N-cadherin, β-catenin, Vimentin, MMP2, MMP9 were down-regulated, and E-cadherin was up-regulated in MOXD1 knockdown cells (Fig. 4F, H).

### Blocking MOXD1 induces cell apoptosis, mitochondrial membrane potential drop, and ROS generation in GBM cells

**Fig. 5:**
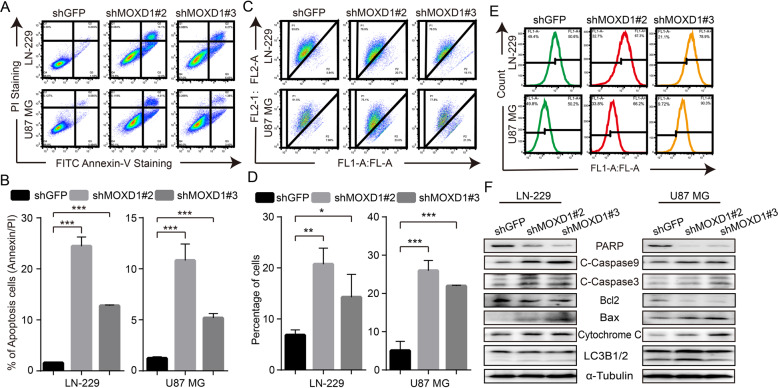
Blocking MOXD1 induces cell apoptosis, mitochondrial membrane potential drop and ROS generation in GBM cells. **A** Apoptosis of MOXD1 knockdown cells was assessed by flow cytometry. **B** Statistics of apoptosis of MOXD1 knockdown cells. **C** mitochondrial membrane potential of MOXD1 knockdown cells was assessed by flow cytometry. **D** Statistics of mitochondrial membrane potential drop of MOXD1 knockdown cells. **E** ROS level MOXD1 knockdown cells was was assessed by flow cytometry. **F** Weatern blot assay was used to deterct mitochondrial apoptosis-related protein.

When MOXD1 was knockdown in GBM cells, we observed that some dead cells were suspended in the medium. Therefore, Annexin V-FITC kit was used to detect cell apoptosis. The results showed that knockdown of MOXD1 can cause obvious apoptosis in GBM cells (Fig. 5A, B). To explore the mechanism of apoptosis, we used JC-1 kit to detect the state of mitochondria. We found that knockdown of MOXD1 can significantly damage and cut down mitochondrial membrane potential of GBM cells (Fig. 5C, D). Moreover, mitochondrial membrane potential drop is closely related to ROS generation in cells. Therefore, after the mitochondrial membrane potential is destroyed, the level of ROS in the GBM cells also rises significantly (Fig. 5E). At the same time, we detect mitochondrial apoptosis pathway related protein PARP, C-Caspase9, C-Caspase3, Bax, Bcl2, Cytochrome C. The results showed that knockdown of MOXD1 can promote cell apoptosis through the mitochondrial apoptotic pathway, and apoptosis has nothing to do with autophagy (Fig. 5F).

### Blocking MOXD1 can trigger ER stress to induce GBM cell apoptosis

**Fig. 6:**
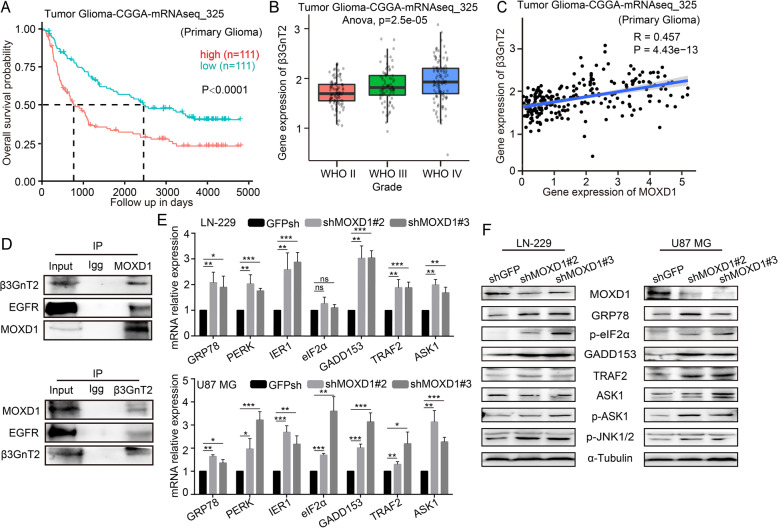
Blocking MOXD1 can trigger ER stress to induce GBM cell apoptosis. **A** Results of the Kaplan–Meier analysis of progression-free survival and the log-rank test *p* values are indicated for tumor glioma-CGGA-mRNAseq-325. **B** Box plot of β3GnT2 expression levels in grade II–IV gliomas in tumor glioma-CGGA-mRNAseq-325. **C** Correlation between MOXD1 and β3GnT2 form tumor glioma-CGGA-mRNAseq-325. **D** IP proved the protein interaction between MOXD1 and β3GnT2. **E** The expression of ER stress-related genes was detected using qRT-PCR. **F** The expression of ER stress-related proteins was detected using Western blotting.

In order to explore the cause of apoptosis induced by MOXD1 knockdown, we explored the functions of MOXD1 on the ER. we analyzed the survival data form the databases (Tumor Glioma-CGGA-mRNAseq-325). The database showed that high β3GnT2 expression is strongly associated with poor survival (Fig. 6A). Kaplan–Meier analysis indicated that β3GnT2 expression level was positively associated with GBM grade (Fig. 6B). Form the database, we found a significant correlation between MOXD1 and β3GnT2 (Fig. 6C). Moreover, the result of Co-immunoprecipitation showed that there is protein interaction between MOXD1 and β3GnT2, and both MOXD1 and β3GnT2 can also bind to epidermal growth factor receptor (EGFR) (Fig. 6D). β3GnT2 is a glycosylation modifying enzyme. Therefore, MOXD1 knockdown may affect the glycosylation of some proteins, causing some unfolded and misfolded proteins to appear on the ER. The results of qRT-RCR suggested that knockdown of MOXD1 induced ER stress and activated the expression of ER stress-related genes (Fig. 6E). Further, WB result once again proved that knockdown of MOXD1 activated the ER stress-induced apoptosis (Fig. 6F). These results indicated that MOXD1 may modify β3GnT2, which in turn affects the glycosylation of some proteins.

## Discussion

GBM is a common primary malignant brain tumor in the cranial cavity accounting for 45.2% of malignant primary brain and central nervous system tumors [13], with a median survival of 12–15 moths and a 5-year survival rate of <5% [14]. Therefore, more and more studies in recent years have tried to reveal the mechanism underlying the GBM cell growth and metastasis. In this study, the levels of MOXD1 were found to be significantly greater in GBM tissues than in adjacent normal tissues. Moreover, higher levels of MOXD1 were associated with higher relapse and overall survival rates in patients with GBM. Subsequently, we investigated the function and mechanism of action of MOXD1 during the development of GBM in vitro and in vivo. The result showed that the knockdown of MOXD1 suppressed GBM cell viability, proliferation, migration and invasion, and promoted G2/M phase cell cycle arrest and cell apoptosis.

Aberrant glycosylation plays a fundamental role in key pathological steps of tumor development and progression [15]. Glycosylation is the process of adding sugars to proteins or lipids under the control of enzymes, which occurs in the ER. Under the action of glycosyltransferase, the sugar is transferred to the protein, and the amino acid residues on the protein form glycosidic bonds [16]. N-acetylglucosaminyltransferases which catalyze the initial branching and elongation reactions involved in the synthesis of O-linked oligosaccharide chains, and make the protein undergo glycosylation modification [17]. Some studies have shown that N-glycosylation on glycosyltransferases is required for their proper-folding and/or enzymatic activities [18, 19]. The study showed that the knockdown of N-acetylglucosaminyltransferase-II suppresses tumorigenicity of neuroblastoma cell [9]. The deletion of UDP-N-acetylglucosamine:alpha-6-d-mannoside beta1,2-N-acetylglucosaminyltransferase II can cause the failure of glycosylation modification of some proteins in the cell [20]. In addition, there are reports stating that β3GnT2, a polylactosamine synthase, regulates glycosylation of EGFR in human hepatocellular carcinoma cells [21]. In this study, we proved that MOXD1 can interact with β3GnT2 protein, both MOXD1 and β3GnT2 can also bind to EGFR. This indicated that MOXD1 may be able to assist and modify β3GnT2 and enable β3GnT2 to perform glycosylation modification functions. Therefore, MOXD1 knockdown affected the function of EGFR, which may also be the reason why the brain of mice injected with shMOXD1 GBM cells was significantly smaller than that of mice injected with shGFP GBM cells. In addition, the knockdown of MOXD1 can also inhibit the glycosylation process of other proteins modified by β3GnT2. As a result of this, the knockdown of MOXD1 will lead to the accumulation of some unfolded and misfolded proteins in the ER, which will cause endogenous stress in the cells.

The perception and response to endogenous or exogenous stress is an important component of cell physiology. Certain evidences show that the ER can initiate the cell’s response to endogenous or exogenous stress [22]. The ER synthetic proteins should be properly folded, glycosylated, and disulfide-bonded to form functional proteins, and ER–mitochondria contact sites is an emerging intracellular signaling [23]. Thus, ER stress pathway has an important role in the development of cancer [13]. Here, knockdown of MOXD1 in GBM cells increased GRP78, subsequent PERK/eIF2α/GADD153 signaling and Ire1α/TRAF2/p-ASK1/p-JNK1/2 signaling. Additionally, we also found that the levels of C-Caspase9, C-Caspase3, Bax, Cytochrome C, and ROS are increased, the mitochondrial membrane potential decreased. These observations suggest that MOXD1 knockdown-mediated ER–Mitochondria pathway may contribute to cancer suppression and apoptosis. MOXD1 is located on the ER of endocrine or non-endocrine cells. MOXD1 may maintain the ER homeostasis under normal circumstances, and may be related to the post-translational protein modification. Under MOXD1 knockdown, the ER homeostasis maybe had broken, which caused the accumulation of unfolded or misfolded proteins in the lumen of the ER. Molecular chaperones such as GRP78 dissociated from the UPR sensors, resulting in their activation. Therefore, we will further explore this phenomenon. At the same time, the relationship between MOXD1 and ER stress in GBM also needs further investigation.

## Materials and methods

### Cell lines, antibodies, and reagents

The normal SVGP12 cell and all GBM cells lines were originally purchased from American Type Culture Collection (ATCC, Rockville, MD, USA). MOXD1 (bs-17733R), GRP78 (bs-1219R), p-eIF2α (bs-4842R), ASK1 (bs-1425R), p-ASK1 (bs-3031R), GADD153 (bs-8875R), TRAF2 (bs-1213R), p-JNK1/2 (bs-4163R), Bax (bs-0127R), Bcl-2 (4563R), Cytochrome C (bs-0013R) were purchased from BOSS (Beijing, China). ATF6 (65880M), Apoptosis Antibody Sampler Kit (9915T), Cell cycle Antibody Sampler Kit (17498T) and the Epithelial–Mesenchymal Transition Antibody Sampler Kit (9782) were purchased from Cell Signaling Technology (Shanghai, China). Lc3B1/2 (14600-1-AP) was purchased from Proteintech (Wuhan, China). Apoptosis kit, JC-1 kit, and ROS kit were purchased from Beyotime Biotechnology. Dulbecco’s modified Eagle’s medium (DMEM), fetal bovine serum (FBS), and penicillin and streptomycin were purchased from BD Becton, Dickinson and Company.

### Transfection and infection

GFP-special interference fragment and MOXD1-special interference fragment (shMOXD1#2:5′-CCGGCCATACTTTGATCTGGTAAATCTCGAGATTTACCAGATCAAAGTATGGTTTTTG-3′ and shMOXD1#3: 5′-CCGGACTAAGCACCAGGAGTGAAATCTCGAGATTTCACTCCTGGTGCTTAGTTTTTTG-3′) was purchased from Sangon and inserted into the pLKO.1 vector that was purchased Addgene (Beijing, China). The procedure of transfection and infection was reference to a report [24].

### RNA extraction, reverse transcription (RT) and real-time PCR (qPCR)

Total RNA extraction, reverse transcription and real-time PCR were reference to a report [25]. The gene names, primer sequences and amplicon sizes are listed in Supplementary Table 1.

### Cell proliferation assay (MTT assay)

The proliferation rate of GBM cells is detected by MTT assay. To test the viability of the cells, we cultured 1000 cells in a 96-well plate for 7 days. Change the medium on the fourth day. MTT analysis is performed on Day 1, Day 3, Day 5 and Day 7, according to the instruction. The experiment was repeated three times independently.

### BrdU assay

Bromodexyuridine (BrdU) assay can detect the rate of cell DNA replication. In order to BrdU staining, we cultured 10,000 cells in a 24-well plate for 2 days. Then adding BrdU (Sigma) to the medium and incubating for 40 min. Subsequently, the cells were washed three times with PBS and fixed with 4% PFA for at least 15 min. After treatment with 2 M hydrochloric acid and 0.1% Triton-100, the cells were blocked with 5% goat serum for 1 h at room temperature, and then incubated overnight with BrdU antibody. After washing the cells with PBS three times, incubating the Alexa flour^*^ 594 secondary antibody for 1 h. DAPI (300 nM) stains the cell nucleus for 20 min. Selecting at least 10 areas to take pictures under the microscope. The experiment was repeated three times independently.

### Flow cytometry

For cell cycle assay, at least 1 × 10^7^ cells fixed with 70% ethanol at 4 °C for 48 h. After washing the cells with PBS three times, the cells incubated with PI (propidium iodide) and RNase at 37 °C for 30 min, and were analyzed by Flow cytometer (BD c6, USA). For cell apoptosis assay, JC-1 assay and reactive oxygen species (ROS) analysis, at least 1 × 10^6^ cells were incubated with Annexin V-FITC/PI kit (YE&SEN), JC-1 kit and ROS assay kit, according to the instruction, and were analyzed by Flow cytometer. The experiment was repeated three times independently.

### Scratch, invasion, migration assay

The cells used for scratches were flattened into a six-well plate. After the cells were overgrown in the six-well plate, they were replaced with serum-free DMEM and cultured for 12 h before scratch assay. After scratching, the cells were cultured in 1% FBS DMEM for 48 h, and were photographed at 0, 24, and 48 h. Transwell chamber was used to detect cell invasion and migration. Matrigel was purchased from BD biosciences, and was used in invasion. 10% FBS DMEM as an inducer is placed under the chamber, and the cells cultured with 1% FBS DMEM is placed in the chamber. After culturing for a period of time, the cells were fixed by 4% PFA for 15 min, and were stained by gentian violet (purchased from Beyotime Biotechnology) for 15 min. After removing the cells in the chamber with absorbent cotton, selecting at least 10 areas to take pictures under the microscope. The experiment was repeated three times independently.

### Soft agar assay

In order to explore the in vitro cloning ability of GBM cells, we configured 2× DMEM medium and 1.2% agar agarose. 2× DMEM and 1.2% agarose were placed in a six-well plate at a ratio of 1:1. 1000 cells per well, placed in 1× DMEM medium, and 2× DMEM and 1.2% agarose on the upper layer of the six-well plate at 2:1:1. After culturing for 18–25 days, colonies was photographed under the microscope.

### Western blot analysis

The cells and tissues were lysed by PIRA and PMSF (purchased from Beyotime Biotechnology). The protein was separated by SDS–PAGE, and was transferred into PVDF membranes. These membranes were incubated separately with primary antibodies overnight at 4 °C, and then were incubated with HRP-linked secondary antibodies for 1.5 h at room temperature. Ultimately, the membranes were exposed by the detection system (ChemiScope 6000, Shanghai, China).

### Xenograft assay

Four-week-old female NOD/SCID mice were purchased and housed in an SPF room that was maintained at a constant temperature and humidity. Human GBM (U87 MG) cells (1 × 10^5^ cells) stably transfected with shGFP, shMOXD1, were injected slowly into mouse intracranial. At the termination of the experiment, the tumors were collected, processed and analyzed as described previously. Randomization and single blinding were used for measurement. Finally, the tumors were collected and photographed for subsequent immunohistochemical staining. All studies were approved by the Animal Care and Use Committee of Southwest University, and carried out in conformity to the Guide for the Care and Use of Laboratory Animals (Ministry of Science and Technology of China, 2006).

### Immunohistochemistry and hematoxylin–eosin staining

Immunohistochemistry staining and hematoxylin–eosin staining were reference to a report [14].

### Patient data analysis and patient tumor tissues

Patient data and gene expression data were obtained from Chinese Glioma Genome Atlas (CGGA), Gene Expression Profiling Interactive Analysis (GEPIA) and R2: Genomics Analysis and Visualization Platform. Patient clinical GBM samples come from the first affiliated hospital of chongqing medical university. Tissue analysis was allowed by the Ethics Committee of Southwest University of China. Written informed consent to participate was provided by all the patients.

### Statistical analysis

All experiments were carried out in triplicates, and the quantitative data are expressed as mean ± SD. A two-tailed Student’s *t* test was performed to calculate the significance in an interval of 95% confidence level. A probability (*p*) value of < 0.05 was considered statistically significant. *p < 0.05, **p < 0.01, ***p < 0.001 indicate different degrees of statistical significance as noted in the figures.

## Supplementary information

Supplementary Figure and table

Supplementary Figure.1

Original Data --scratches and transwell

Original Data --IHC and HE

Original Data --miceoscopic and BrdU

Original Data -Westtren blot

Original Data --cell cycle, apoptosos, JC-1, ros

Supplementary Table 1

## Data Availability

All data and material in the study are available when requested.
