# Supplementary material for: MOXD1 knockdown suppresses the proliferation and tumor growth of glioblastoma cells via ER stress-inducing apoptosis
Source: Cell Death Discov. 2022 Apr 7;8:174. doi: 10.1038/s41420-022-00976-9 (PMC8991257; doi:10.1038/s41420-022-00976-9)
Supplement: Supplementary file 1 — Supplementary Figure and table [file 41420_2022_976_MOESM1_ESM.docx]

**Supplementary Figure.1 The relationship between MOXD1 expression and survival, age, gender, IDH mutation, 1p/19q co-deletion** **of patients with GBM. A** Results of the Kaplan-Meier analysis of progression-free survival and the log-rank test *p* values are indicated for Tumor Glioma-French-284. **B** The relationship between MOXD1 expression and patient's IDH mutation. **C** The relationship between MOXD1 expression and patient's 1p/19q co-deletion. **D** The relationship between MOXD1 expression and patient's age. **E** The relationship between MOXD1 expression and patient's gender.

**Supplementary Table1**  **Primer sequences, amplicon sizes in base pairs (bp) and reference GenBank accession numbers**
