# Supplementary figures and images for: MOXD1 knockdown suppresses the proliferation and tumor growth of glioblastoma cells via ER stress-inducing apoptosis
Source: Cell Death Discov. 2022 Apr 7;8:174. doi: 10.1038/s41420-022-00976-9 (PMC8991257; doi:10.1038/s41420-022-00976-9)

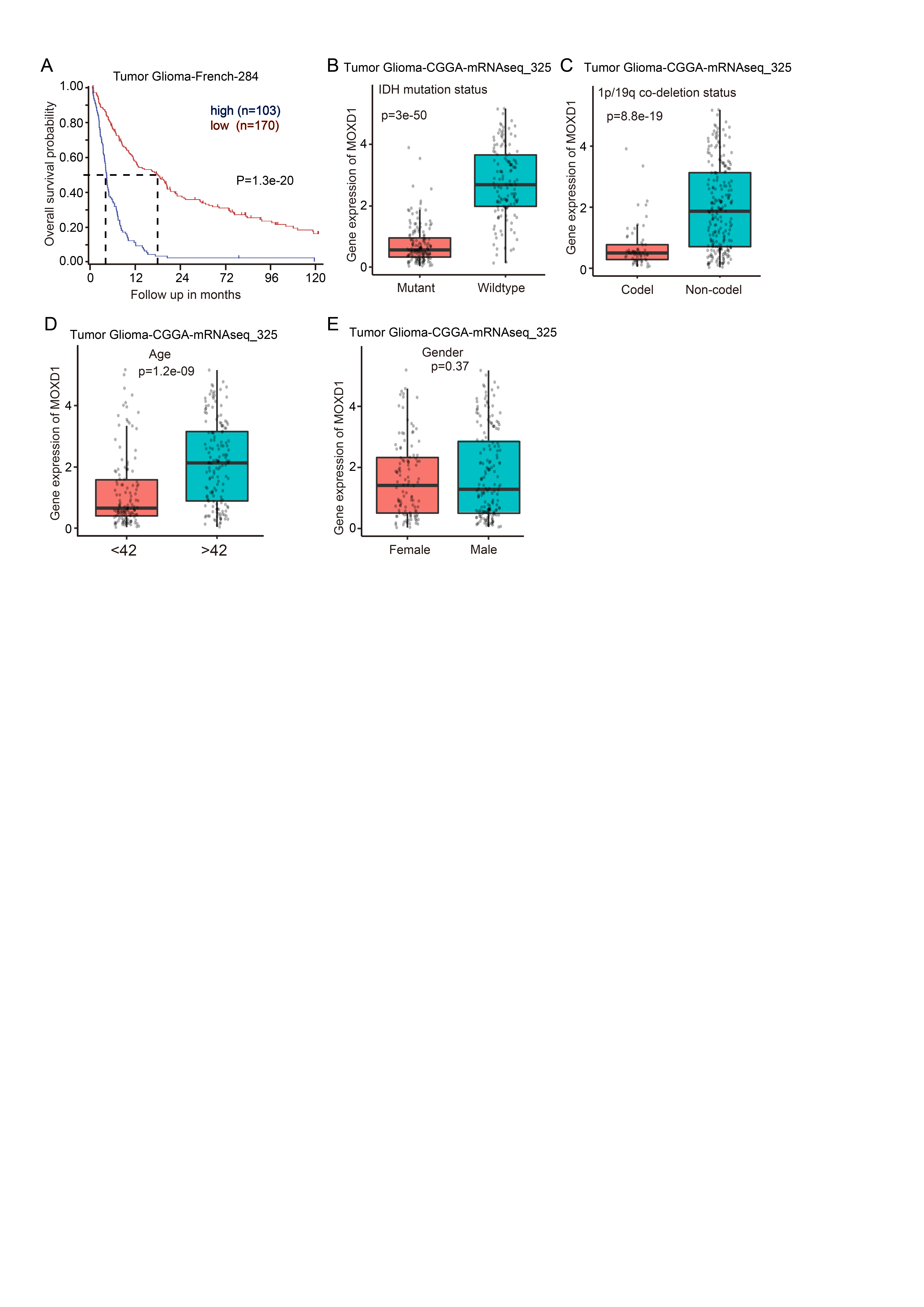

Supplement: Supplementary file 2 — Supplementary Figure.1 [file 41420_2022_976_MOESM2_ESM.jpg]
