# Supplementary material for: MOXD1 knockdown suppresses the proliferation and tumor growth of glioblastoma cells via ER stress-inducing apoptosis
Source: Cell Death Discov. 2022 Apr 7;8:174. doi: 10.1038/s41420-022-00976-9 (PMC8991257; doi:10.1038/s41420-022-00976-9)
Supplement: Supplementary file 8 — Supplementary Table 1 [file 41420_2022_976_MOESM8_ESM.docx]

Supplementary Table1 Primer sequences, amplicon sizes in base pairs (bp) and reference GenBank accession numbers

| Gene name | Entrez gene | Primer sequence(5’-3’) | Product length(bp) | Accession number(NCBI) |
| --- | --- | --- | --- | --- |
| Monooxygenase DBH Like 1 | MOXD1 | F:GCATCAGGCTGCGTCATTTTC  R:TCGTGTTGTAGCGACTCAG | 153 | [NM_015529.4](https://www.ncbi.nlm.nih.gov/nuccore/NM_015529.4) |
| Heat Shock Protein Family A (Hsp70) Member 5 | GRP78 | F:GAACGTCTGATTGGCGATGC  R:ACCACCTTGAACGGCAAGAA | 143 | [NM_005347.5](https://www.ncbi.nlm.nih.gov/nuccore/NM_005347.5) |
| Protein kinase R‑like endoplasmic reticulum kinase | PERK | F:TGCATATAGTGGAAAGGTGAGGT  R:GAGGTCCGACAGCTCTAACAG | 134 | [XM_017005376.2](https://www.ncbi.nlm.nih.gov/nuccore/XM_017005376.2) |
| Inositol‑requiring enzyme 1 | IER1 | F:TGTACGACACCAAAACCCGA  R:CACAGGGGAGGCGTAGTTTT | 185 | [NM_002922.4](https://www.ncbi.nlm.nih.gov/nuccore/NM_002922.4) |
| Eukaryotic Translation Initiation Factor 2 Subunit Alpha | eIF2α | F:ACTACGACAACCCTGGAGAGA  R:CTGCCTCGCAAGTTCAGTCT | 156 | [NM_004094.5](https://www.ncbi.nlm.nih.gov/nuccore/NM_004094.5) |
| DNA Damage Inducible Transcript 3 | GADD153 | F:CTGGAAAGCAGCGCATGAAG  R:GGTGCAGATTCACCATTCGG | 159 | [NM_001195053.1](https://www.ncbi.nlm.nih.gov/nuccore/NM_001195053.1) |
| TNF Receptor Associated Factor 2 | TRAF2 | F:GGAGGCATCCACCTACGATG  R:GGGAGAAGATGGCGGGTATG | 101 | [XM_011518976.3](https://www.ncbi.nlm.nih.gov/nuccore/XM_011518976.3) |
| Mitogen-Activated Protein Kinase Kinase Kinase 5 | ASK1 | F:GCAGCCGTCCTTGTTTTACC  R:GTGACTGCAGAGAGTCCGAG | 101 | [NM_005923.4](https://www.ncbi.nlm.nih.gov/nuccore/NM_005923.4) |
| Glyceraldehyde-3-Phosphate Dehydrogenase | GAPDH | F:ATTCCACCCATGGCAAATTCC  R:GACTCCACGACGTACTCAGC | 145 | [NM_002046.7](https://www.ncbi.nlm.nih.gov/nuccore/NM_002046.7) |
